# The Impact of Collection Protocol on the Yield and Purity of Mesenchymal Stem Cell‐Derived Extracellular Vesicles Isolated From Serum‐Free Media

**DOI:** 10.1002/biot.70264

**Published:** 2026-07-02

**Authors:** Jolene Phelps, Sara Hassanpour Tamrin, Neil A. Duncan, Arindom Sen

**Affiliations:** ^1^ Pharmaceutical Production Research Facility Schulich School of Engineering, University of Calgary Calgary Alberta Canada; ^2^ Department of Biomedical Engineering, Schulich School of Engineering University of Calgary Calgary Alberta Canada; ^3^ McCaig Institute For Bone and Joint Health, Cumming School of Medicine University of Calgary Calgary Alberta Canada; ^4^ Musculoskeletal Mechanobiology and Multiscale Mechanics Bioengineering Lab, Schulich School of Engineering University of Calgary Calgary Alberta Canada; ^5^ Department of Civil Engineering, Schulich School of Engineering University of Calgary Calgary Alberta Canada; ^6^ Department of Chemical and Petroleum Engineering, Schulich School of Engineering University of Calgary Calgary Alberta Canada

**Keywords:** bioprocessing, cell confluence, exosomes, extracellular vesicles, mesenchymal stem cells, microvesicles, serum‐free medium

## Abstract

The immunomodulatory and regenerative effects of mesenchymal stem cell (MSC) extracellular vesicles (EVs) have spurred the development of strategies to manufacture these EVs as therapeutics. Clinical‐grade EV manufacturing requires defined serum‐free media (SFM) to reduce heterogeneity and improve batch reproducibility, yet standard protocols for collecting EVs from SFM are lacking. A primary concern is the co‐isolation of SFM‐derived proteins with EVs during separation, which can interfere with the characterization of the collected EVs, and their subsequent application. In this study, we evaluated how removing proteins from SFM affects EV yield and purity, and examined how collection timing and cell confluence influence EV yield and proteomic profile. A defined SFM (PPRF‐msc6) was compared to (i) an ultracentrifuged medium (PPRF‐msc6 after overnight ultracentrifugation), and (ii) a starvation medium (PPRF‐msc6 without albumin and fetuin). MSC growth and viability were reduced in starvation medium, but were not adversely impacted when using ultracentrifuged medium. Compared to unmodified PPRF‐msc6, ultracentrifuged medium improved EV purity but lowered EV yield. MSC confluence impacted the proteomic profile of EV fractions, demonstrating the importance of determining when EVs are collected during the culture period. Defining protocols for MSC‐EV collection contributes to standardization within the rapidly growing EV field, and informs the development of clinically relevant bioprocesses.

## Graphical Abstract and Lay Summary

Mesenchymal stem cells (MSCs) were cultured in standard, ultracentrifuged (UC), or starvation serum‐free media to evaluate how medium influences extracellular vesicle (EV) yield and purity. Culture in UC medium improved EV purity but reduced yield. Changes in cell confluence altered EV composition, highlighting key variables for standardized clinical MSC‐EV manufacturing.

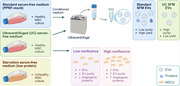

**Abbreviations**
ANG: Angiopoietin
bFGF: Basic fibroblast growth factor
BMP: Bone morphogenic protein
BSA: Bovine serum albumin
CD105: Endoglin
CM: Conditioned medium
DPBS: Dulbecco's phosphate‐buffered saline
EGF: Epidermal growth factor
EV: Extracellular vesicle
EVCM: Extracellular vesicle collection medium
FGF‐1: Acidic fibroblast growth factor
FST: Follistatin
G‐CSF: Granulocyte colony‐stimulating factor
HB‐EGF: Heparin‐binding epidermal growth factor
HGF: Hepatocyte growth factor
HSA: Human serum albumin
IL: Interleukin
MSC: Mesenchymal stem cell
PLGF: Placental growth factor
PPRF: Pharmaceutical Production Research Facility
SFM: Serum‐free medium
SP‐IRIS: Single particle interferometric reflectance imaging sensor
SS: Serum starvation
TEM: Transmission electron microscopy
UC: Ultracentrifugation; Ultracentrifuged; Ultracentrifuge
VEGF: Vascular endothelial growth factor

## 1 Introduction

Mesenchymal stem cells (MSCs) are a heterogeneous population of multipotent cells with immunomodulatory and regenerative properties [1]. Increasing evidence suggests that their therapeutic efficacy may be attributed to paracrine mechanisms via secreted extracellular vesicles (EVs) [1, 2]. EVs are nanoparticles with a lipid bilayer membrane enclosing bioactive signaling cargo, such as proteins, lipids, and nucleic acids, that can elicit changes in the behavior and function of recipient cells. They are widely recognized for their role in cellular communication, and are now seen as promising therapeutic candidates and disease biomarkers [3]. Compared to cell therapies, the use of MSC‐EVs as therapeutics overcomes several hurdles to clinical translation; namely, their non‐living nature, which enables accurate dosing and quality control while easing storage and transport compared to viable cells [2, 4]. The regenerative properties of MSC‐EVs have been widely reported [5]; however, there is a need to produce MSC‐EVs in a clinically relevant manner and enhance process repeatability without compromising MSC health and defining qualities.

EV attributes are linked to the characteristics of the cells from which they are derived. When culturing MSCs, the medium used and the density of the resulting MSC monolayer (i.e., confluence) are known to impact the quality of the cells, suggesting that these variables may also influence the attributes of EVs produced by those cells [2]. MSCs are most commonly grown in a basal medium, such as Dulbecco's Modified Eagle's Medium (DMEM), that has been supplemented with serum, which is problematic from an EV collection standpoint, as serum naturally contains a high concentration of endogenous EVs which end up co‐isolating with MSC‐EVs, thereby serving as a major contaminant [6, 7, 8]. To address this issue, researchers now commonly grow MSCs in a serum‐containing medium until they reach 60%–90% confluence, at which point the serum‐containing medium is replaced by a serum‐free EV collection medium (EVCM). The cells remain in culture but now secrete EVs into this EVCM. The EVCM is typically either (i) the same basal medium used during the cell growth phase, but now, instead of containing serum, is supplemented with serum that has been EV‐depleted by overnight ultracentrifugation, or (ii) a starvation medium that consists of the basal medium without serum. The EVCM is recovered after 24–48 h, at which point it is termed conditioned medium (CM), and processed to isolate the EVs [6].

The use of serum‐containing media, even if pre‐cleared of EVs, is clinically undesirable due to the undefined nature of serum, which can lead to high batch‐to‐batch differences in medium composition and cause significant variation in EV population characteristics between batches [9]. Moreover, EV‐depletion from serum is not 100% efficient, leaving behind a significant number of EVs while removing other components, which may adversely impact cell expansion and EV production [10]. Serum is also commonly derived from animals and carries risks of prion and viral transmission, and may result in adverse reactions from components retained within the cells or cell products after transplantation [11]. Chemically defined serum‐free medium (SFM) formulations have demonstrated increased EV secretion compared to standard DMEM [12], and can be tuned to better support the production of EVs that exhibit increased therapeutic potential [13]. Our lab previously developed PPRF‐msc6, a fully published SFM [14] that supports the expansion of MSCs in a rapid and consistent manner, and could contribute to reduced heterogeneity and increased functionality of MSC‐EV populations [14, 15, 16, 17].

Notably, commonly used EV production protocols have been optimized for cells expanded in serum‐containing medium and are not specific to MSC populations, which are more heavily influenced by cell confluence. While PPRF‐msc6 (and other SFM) does not contain EVs, it does contain a significant amount of protein that may co‐isolate with EV populations [18]. These co‐isolated proteins can serve as contaminants that significantly influence subsequent EV characterization and functionality studies [19, 20]. To address this issue, an SFM can be replaced by an EVCM devoid of the protein fraction that would co‐isolate with EV populations. However, an important consideration in this scenario is that these proteins may be essential for MSC survival, maintenance, and functionality. Thus, the impact of using such a protein‐reduced EVCM on MSC growth and surface marker expression, and on the characteristics of the EV populations produced during the collection period needs to be determined.

There is also evidence that the functionality of MSCs is impacted by cell confluence [21, 22]. It is generally accepted that the initial plating density of MSC cultures is not crucial to maintain well‐defined multipotent MSC populations. However, longer times in culture, and the resulting higher confluence of MSCs, have been reported to lower their expression of stemness genes, reduce viability, increase doubling times, and reduce their angiogenic and migratory properties [21, 22, 23, 24, 25]. MSC harvest at 80% confluence has been proposed to optimize MSC characteristics while still enabling sufficient growth [22]. There is limited literature on the effect of MSC confluence on the EVs they produce. Although cell confluence has been reported to impact EV production yield, with greater numbers of EVs per cell being produced at lower confluence when cells are rapidly proliferating [26], total EV production is limited at low confluence because of the smaller number of cells. No differences in the vascularization bioactivity of EVs were reported between those derived from low and high confluence MSC cultures when compared on the basis of equal protein [26], which raises the question of whether EVs produced from MSCs at high confluence retain the same composition of protein as those produced from MSCs at low confluence.

The aims of this study were to evaluate (i) if removing protein contaminants from PPRF‐msc6 medium prior to being used for EV collection affects MSC proliferation, surface marker expression, and corresponding EV recovery and purity; and (ii) if MSC confluence alters EV yield and the proteomic profile of the EV fraction. Regarding the proteomic profile, changes in proteins related to angiogenesis were evaluated, given their relevance to regenerative medicine applications such as stroke, traumatic brain injury, and wound healing, where the induction of blood vessel formation is critical for restoring blood flow and supporting functional recovery. Better defining the protocols used for EV collection will contribute to the development of standards in the developing EV field, enable comparison of results across research groups thereby accelerating knowledge accumulation, support the development of robust EV bioprocesses, and facilitate the clinical utilization of MSC‐EVs in therapeutic applications.

## 2 Materials and Methods

### 2.1 MSC Culture

Ethically approved human adipose‐derived MSC populations (University of Calgary Health Research Ethics Board ID: REB15‐1005) were isolated from abdominal subcutaneous adipose tissue (female, age 20–30, BMI within normal range) and expanded in PPRF‐msc6 as previously described [16]. For experiments where initial seeding density is not listed, MSCs were inoculated at 5000 cells/cm^2^. Cells were passaged using TrypLE Express.

### 2.2 EV Collection

Growth medium was removed from MSC cultures 48–72 h post‐inoculation, and the cells were washed twice with Dulbecco's phosphate‐buffered saline (DPBS). Thereafter, 12 mL of EVCM was added to each T‐75 and recovered after 24–48 h. Ultracentrifuged (UC) EVCM was prepared by ultracentrifuging PPRF‐msc6 medium at 105,000 g for 18.5 h (Beckman Coulter Optima L‐100K, 70 Ti rotor, 38,000 rpm, *k* = 148) and then passing it through a 0.22 µm filter. Starvation (SS) EVCM was prepared as PPRF‐msc6 medium without human serum albumin (HSA) and fetuin.

### 2.3 Flow Cytometry

MSCs were washed with DPBS, stained with 1 µL/mL Live/Dead Fixable Blue Dead Cell Stain (ThermoFisher Scientific, Waltham, MA, USA), and incubated in the dark for 30 min at room temperature. Cells were then blocked on ice in the dark for 20 min. Five µL of each antibody (Table S1) was added to cell aliquots of 5 × 10^5^ cells in 100 µL of blocking buffer and incubated on ice in the dark for 30 min. Cells were washed with DPBS and fixed with 4% paraformaldehyde in DPBS (Santa Cruz Biotechnology Inc., Dallas, TX, USA) in the dark on ice for 15 min. Finally, cells were washed with Flow Cytometry Staining Buffer (R&D Systems, Minneapolis, MN, USA) and stored at 4°C. Samples were run within 72 h on a BD LSR II (BD Biosciences, Franklin Lakes, NJ, USA) flow cytometer, and data analysis was completed using FlowJo V10.8.1.

### 2.4 EV Isolation

Differential ultracentrifugation was used to isolate small EV fractions as previously described [16]. Briefly, expended medium was centrifuged at 2000 g and 4°C for 10 min, then at 10,000 g and 4°C for 30 min, and then diluted 1:1 with DPBS and ultracentrifuged at 105,000 g and 4°C for 2 h (Beckman Coulter Optima L‐100K, 70 Ti rotor, 38,000 rpm, *k* = 148). The pellet containing the EV fraction was re‐suspended in DPBS for transmission electron microscopy (TEM) and single particle interferometric reflectance imaging sensor (SP‐IRIS) analyses, or in RIPA buffer (1x with 10 µL/mL protease inhibitors (EMD Millipore, Burlington, MA)) for protein analyses. All EV fractions were resuspended to a concentration factor of 50x the originating CM (i.e., for 10 mL CM, the EV pellet was resuspended in 200 µL of DPBS or RIPA buffer). Resuspended EV fractions were either used immediately or frozen at −80°C (without freeze‐thaw) for subsequent analyses.

Note: A washing step involving a second round of ultracentrifugation is often reported to eliminate contaminating proteins [27], despite significantly reducing EV yield [28]. In prior experiments, we found a significant reduction in EV yield with no significant increase in EV purity (Figure S1), consistent with other studies using SFM [29].

### 2.5 Protein Quantification

A Pierce BCA Protein Assay Kit (ThermoFisher Scientific, Waltham, MA, USA) was used to measure total protein content in CM and EV samples, as per manufacturer directions. Briefly, 20 µL of each sample or standard was added to 200 µL of working reagent and incubated at 37°C for 30 min. Absorbance was read at 562 nm on a SpectraMax iD3 plate reader (Molecular Devices, San Jose, CA, USA). Angiogenic protein quantification was determined using a Luminex Angiogenesis Discovery Assay (Eve Technologies, Calgary, Canada), measuring angiopoietin (ANG)‐2, bone morphogenic protein (BMP)‐9, epidermal growth factor (EGF), endoglin (CD105), endothelin (ET)‐1, acidic fibroblast growth factor (FGF‐1), basic fibroblast growth factor (bFGF), follistatin (FST), granulocyte colony‐stimulating factor (G‐CSF), heparin‐binding epidermal growth factor (HB‐EGF), hepatocyte growth factor (HGF), interleukin (IL)‐8, leptin, placental growth factor (PLGF), vascular endothelial growth factor (VEGF)‐A, VEGF‐C, and VEGF‐D. For protein measurements, all EVs were lysed by resuspension in 1x RIPA buffer with protease inhibitors (see Section 2.4).

### 2.6 Transmission Electron Microscopy (TEM)

TEM was used to confirm EV morphology and size using a Hitachi H7650 120 kV microscope (Hitachi High‐Tech, Tokyo, JP). Briefly, EVs were adsorbed to formvar‐coated copper mesh grids (Electron Microscopy Sciences, Hatfield, PA) for 30 min, fixed in 2.5% glutaraldehyde for 15 min, washed twice with dH_2_O, stained with 2.6% uranyl acetate, washed twice again with dH_2_O, and dried at room temperature before being imaged at 80 kV.

### 2.7 Single Particle Interferometric Reflectance Imaging Sensor (SP‐IRIS) Imaging

An ExoView R100 (NanoView Biosciences, Boston, MA) SP‐IRIS device was used for particle counts, size, and concentration using EV‐specific (CD81, CD63, and CD9) antibody‐coated microarray tetraspanin chips. EV samples were diluted 1:500, and CM samples were diluted 1:100 with incubation buffer. 50 µL of each sample was incubated at room temperature for 16 h on the chips. The chips were then washed and stained with fluorescently conjugated antibodies CD81, CD63, and CD9 according to manufacturer protocols.

### 2.8 Statistics

Data are presented as mean ± standard deviation. One‐way ANOVA followed by post hoc analysis using the Bonferroni multiple comparisons test was used to compare between conditions in GraphPad Prism 6. Differences were deemed significant for *p* < 0.05. *N* = 3 individual culture flasks and/or EV isolations for all analyses, *p < 0.05, **p < 0.01, ***p < 0.001, ****p < 0.0001.

## 3 Results

### 3.1 MSC Growth and Surface Marker Expression Varies Depending on EV Collection Medium

**FIGURE 1:**
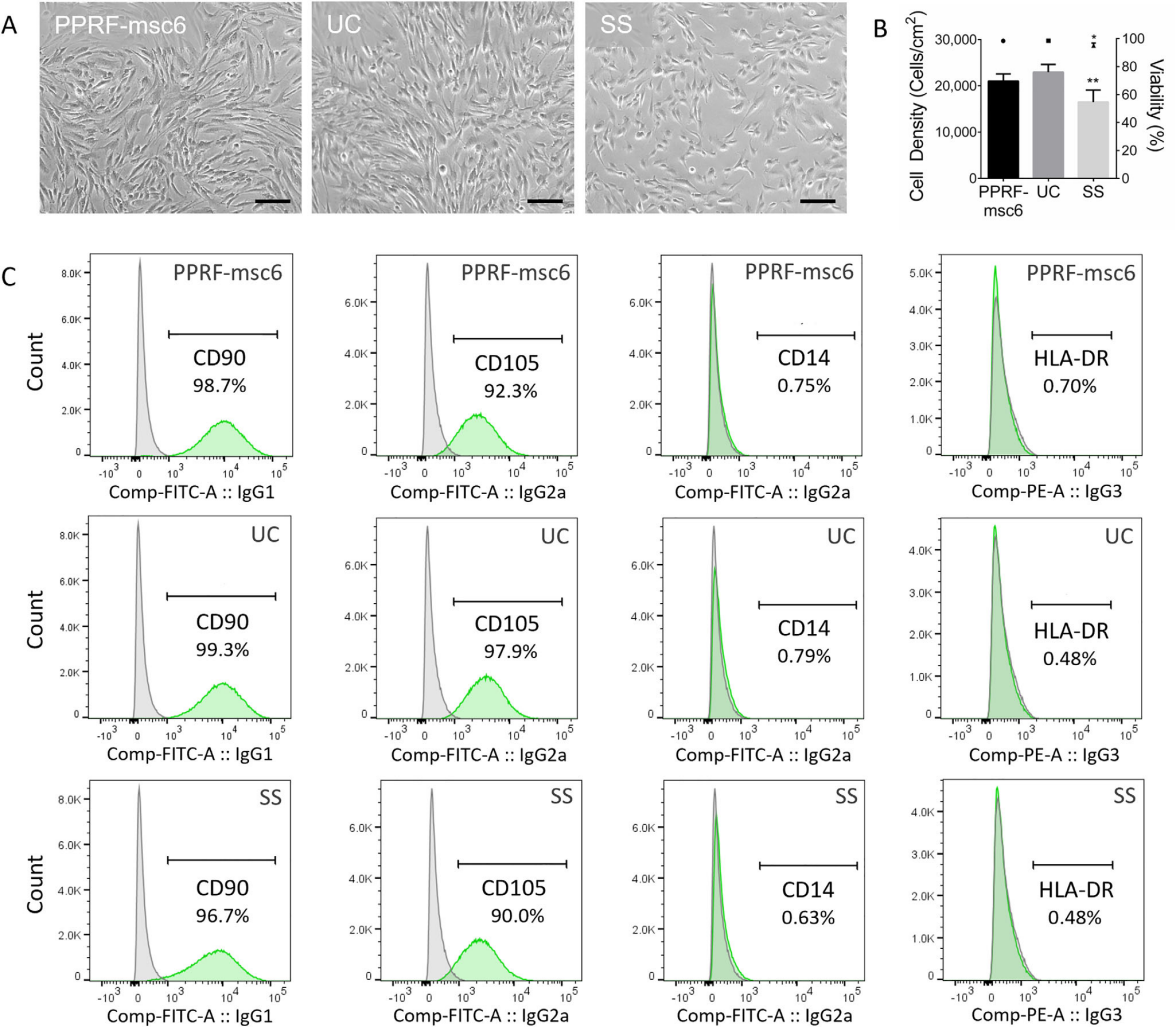
MSC growth and surface maker expression on day 4 following culture in PPRF‐msc6 medium, ultracentrifuged medium (UC), and starvation medium (SS). Cells were grown in PPRF‐msc6 up to day 3, washed twice with DPBS, and then cultured in PPRF‐msc6, UC, or SS medium for an additional 24 h. (A) Phase contrast microscopy images of MSCs on day 4 at the time of harvest, cultured in PPRF‐msc6, and UC and SS media. Scale bar = 100 µm. (B) Cell density at time of harvest. Significance measured relative to PPRF‐msc6 (*N* = 3). (C) Flow cytometry analysis of CD90, CD105, CD14, and HLA‐DR expression of MSCs cultured in PPRF‐msc6, UC, and SS media. Gray represents control, and green represents experimental conditions.

The effect of EVCM on MSC growth, viability, and surface marker expression was performed by culturing MSCs in (i) PPRF‐msc6 medium, (ii) UC medium, and (iii) SS medium. MSCs were cultured for 72 h (∼70% confluence), washed twice with DPBS, and cultured in varying EVCM for 24 h. No change in MSC growth was seen in UC medium, while cell growth and viability were significantly impacted by culture in SS medium (Figure 1A,B). The percentage of CD105‐positive cells was retained only in UC medium and was reduced in PPRF‐msc6 and SS media (92.3% and 90%, respectively, compared to 97.9% in UC medium) (Figure 1C). The percentage of CD90‐expressing cells remained >95%, and CD14 and HLA‐DR‐expressing cells remained <2% for all conditions. Notably, MSCs exhibited high viability at the time of EV collection in both PPRF‐msc6 and UC medium (>96%), and prior work from our lab confirmed that MSCs were still within the exponential growth phase on day 4 at the current inoculation density [30].

To test longer‐term effects of the different media, MSCs were cultured from day 0–3 in each EVCM. Cell density and viability were significantly lower in SS medium, while UC medium reduced proliferation but not viability (Figure S2). No changes in cell expression of CD105, CD90, CD14, or HLA‐DR were seen when comparing culture in PPRF‐msc6 and UC medium.

### 3.2 UC Medium Increases EV Purity but Reduces EV Yield

**FIGURE 2:**
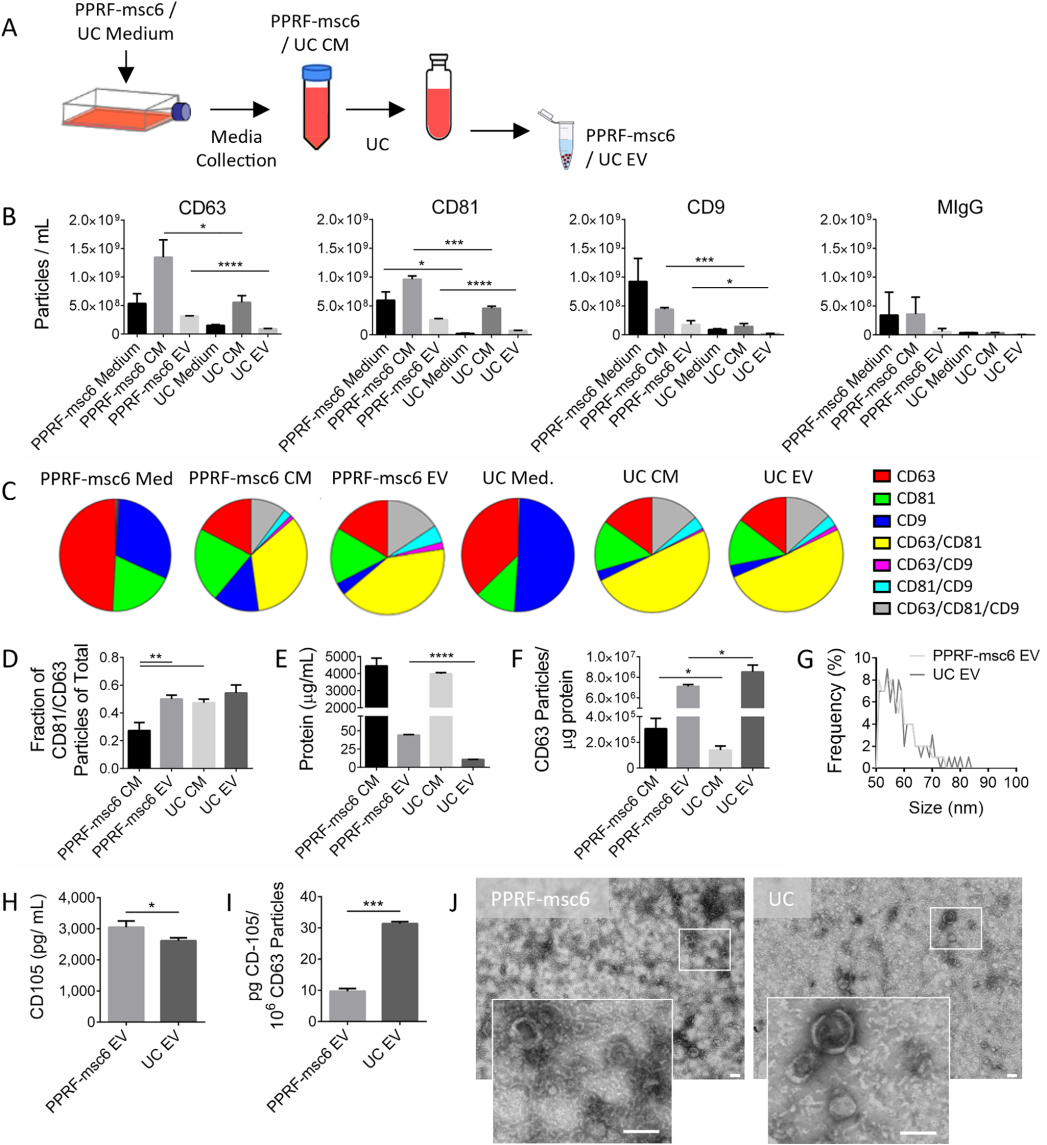
SP‐IRIS and protein analyses of EV fractions isolated from day 4 MSCs cultured in either PPRF‐msc6 or ultracentrifuged PPRF‐msc6 (UC) media for 24 h (*N* = 3). (A) Schematic of the process used for producing and isolating EV fractions. (B) Total particles bound to CD63, CD81, CD9, and MIgG antibodies on microarray tetraspanin chips measured by SP‐IRIS. (C) Colocalization charts for representative samples bound to CD63. (D) Fraction of colocalized CD63/CD81 particles of total particles. (E) Total protein content. (F) CD63 particles per µg protein. (G) Particle size distribution. (H) CD105 concentration. (I) CD105 concentration per 10^6^ CD63 particles. (J) Representative TEM images. Scale bar = 100 nm.

EV yield and purity were evaluated after MSC exposure to PPRF‐msc6 and UC media over a 24 h period via SP‐IRIS analyses of the CM and EV fractions. The total particle concentration was considerably lower in the UC CM and EV fractions compared to the PPRF‐msc6 CM and EV fractions (Figure 2B). Controls of fresh PPRF‐msc6 and UC media were evaluated, and substantial particle counts were seen for PPRF‐msc6 medium, while UC medium exhibited lower particle counts. Prior results found minimal binding of components from the pellet obtained from fresh PPRF‐msc6 medium (Figure S1), indicating antibody binding was primarily to components in the non‐EV protein‐rich fraction. Comparing colocalization profiles, minimal colocalization was seen for fresh PPRF‐msc6 and UC media controls, and the particles in UC CM exhibited a similar colocalization profile as that of UC EVs, with a lower portion of CD9 appearing in the UC CM compared to the PPRF‐msc6 CM (Figure 2C).

Comparing the fraction of co‐localized CD63/CD81 particles to the total, both EV fractions exhibited similar levels of particle co‐localization (Figure 2D); however, the fraction of co‐localized particles was higher for UC CM compared to PPRF‐msc6 CM. The total protein content in UC EVs was significantly lower than that of PPRF‐msc6 EVs (Figure 2E). Comparing the purity of EV fractions (measured by total CD63 particles per µg of protein), the UC EV fraction exhibited higher purity compared to the PPRF‐msc6 EV fraction (Figure 2F). Finally, CD105 concentration was compared in EV fractions, measured from total lysed particles, to evaluate if a change in transmembrane protein content could be found in EVs isolated from PPRF‐msc6 and UC medium. The CD105 concentration per particle was significantly higher in UC EVs (Figure 2I), which could indicate that the transmembrane protein content of EVs changes depending on culture medium. TEM images for both conditions confirmed the presence of EVs (Figure 2J), with a higher abundance of protein seen in PPRF‐msc6 EV fractions (black shading).

### 3.3 EV Recovery for UC Medium Can be Improved by Adding Albumin Prior to EV Isolation

**FIGURE 3:**
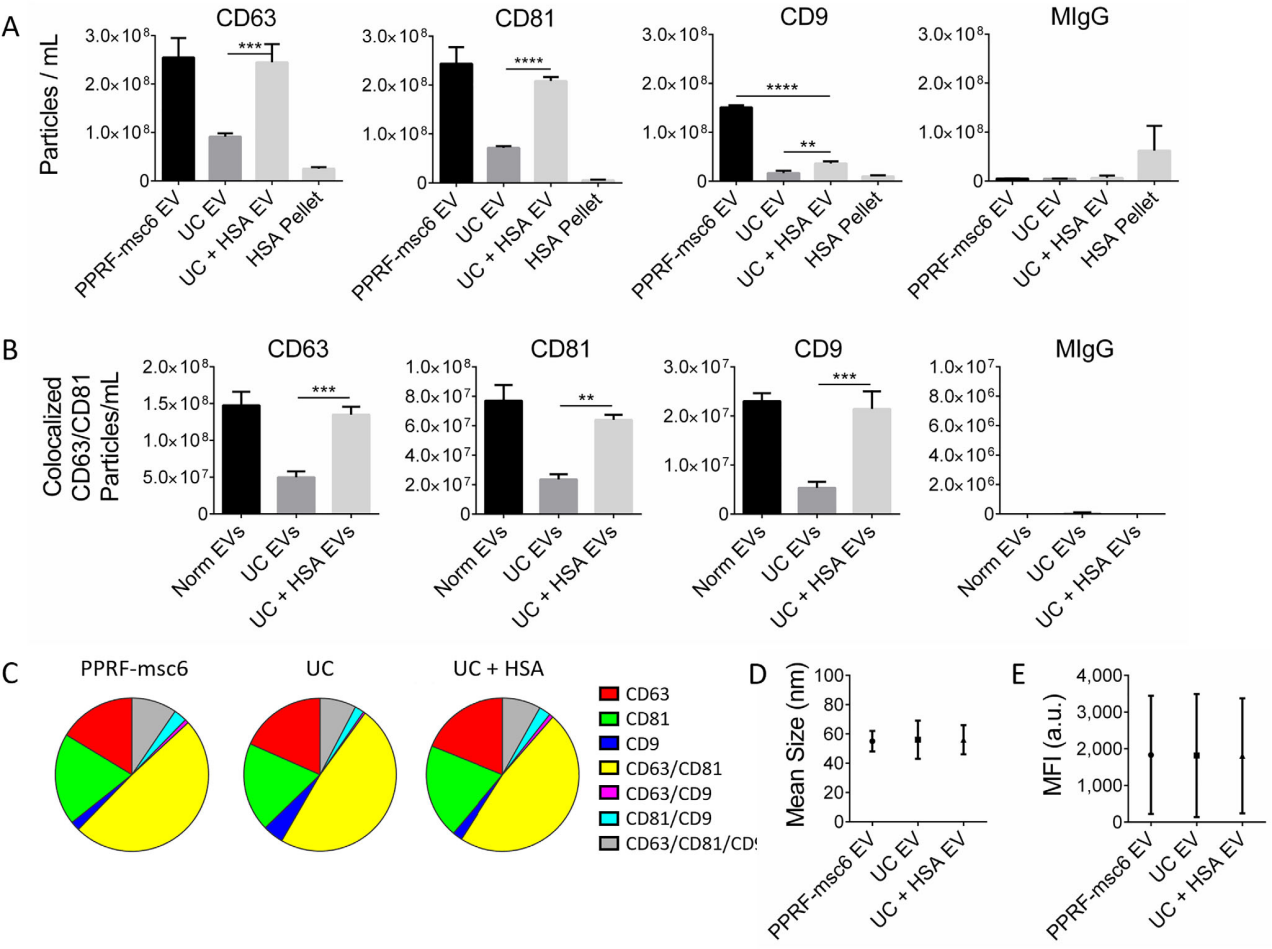
(A) Total particles bound to CD63, CD81, CD9, and MIgG antibodies on microarray tetraspanin chips measured by SP‐IRIS for EV fractions from PPRF‐msc6 CM, ultracentrifuged PPRF‐msc6 (UC) CM, and UC CM with HSA (UC + HSA). (B) Total colocalized CD63/CD81 particles bound to CD63, CD81, CD9, and MIgG antibodies. (C) Colocalization charts for representative samples bound to CD63. D) Mean size and standard deviation for each condition. (E) Mean fluorescent intensity (MFI) per particle (*N* = 3). Significant differences were shown only between UC + HSA EVs—all differences between PPRF‐msc6 and UC‐EVs were significant (*p* < 0.001).

To better understand the difference in EV recovery between EVs isolated from PPRF‐msc6 and UC CM, it was hypothesized that albumin (largely removed during overnight UC) may play an important role in the stability and recovery of EVs during isolation, based on recent findings that the addition of albumin in storage buffers increases EV recovery [31]. To test this, HSA was added directly to UC CM following EV collection, prior to EV isolation, at the same concentration found in fresh PPRF‐msc6 (4% w/v). Total particles bound to CD63, CD81, CD9, and MIgG were then measured for EV fractions isolated from PPRF‐msc6 CM, UC CM, and UC CM supplemented with HSA (UC + HSA) (Figure 3A,B). No increase in non‐specific binding to MIgG was found with the addition of HSA, and there was minimal binding of HSA alone to all antibodies (HSA pellet). CD63 and CD81 positive particle concentrations increased in the HSA‐supplemented UC medium compared to the non‐supplemented UC medium and were equivalent to those found in PPRF‐msc6, indicating that HSA may facilitate particle recovery during UC. The degree of co‐localization was equivalent for all samples (Figure 3C). The measured size and mean fluorescent intensity of these populations were not significantly different, negating any concerns with differing amounts of particle aggregation (Figures 3D,E). The effect of coating UC tubes with albumin was also tested, with no significant differences in recovery found (Figure S3).

### 3.4 EV and Protein Yield Differs With MSC Confluence

To better understand how EV yield and composition change with MSC confluence, two conditions were evaluated: inoculation density and time of EV collection. MSCs were seeded at three different densities (500, 2500, or 5000 cells/cm^2^), and for each case, EVs were collected during three periods: day 2 for 24 h (day 2–3), day 3 for 24 h (day 3–4), and day 2 for 48 h (day 2–4). The use of different inoculation densities enabled the study of MSC‐EVs at differing confluence but collected at the same time point post‐inoculation, aligning with previous studies [26]. The collection of EVs from different time points was further carried out to evaluate if a different concentration or subset of EVs would be produced at different periods during culture.

**FIGURE 4:**
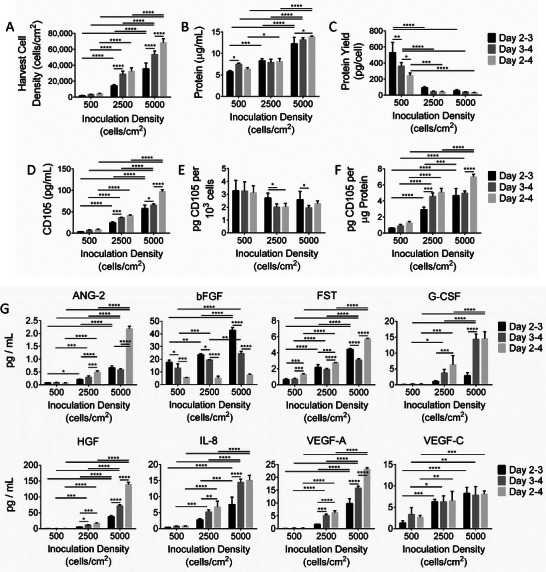
MSC growth and MSC‐EV protein concentrations following culture at differing inoculation densities and different EV collection times (*N* = 3). (A) Cell density at time of EV harvest. (B) Total protein in the EV fraction. (C) Total protein in the EV fraction per cell. (D) CD105 concentration in the EV fraction normalized to per mL of CM. (E) CD105 concentration in the EV fraction per 10^3^ cells. (F) CD105 concentration per total protein in the EV fraction. (G) Total angiogenic protein concentrations in EV fractions. All concentrations are normalized to per mL of CM.

PPRF‐msc6 medium was replaced with UC medium at the beginning of each EV collection period. For cultures inoculated at 5000 cells/cm^2^, day 4 cell densities were significantly higher when the EV collection medium was added on day 2 compared to day 3 (Figure 4A). No significant differences were found for cultures inoculated at lower densities. Total protein content was significantly higher in EV fractions obtained from cultures inoculated at 5000 cells/cm^2^ compared to lower densities, with greater similarity between those inoculated at 500 and 2500 cells/cm^2^ (Figure 4B). Total protein yield (protein/cell) was highest for cells at low confluence (day 2–3 500 cells/cm^2^) with no significant difference between those cultured at 2500 and 5000 cells/cm^2^ (Figure 4C). CD105 was analyzed to evaluate changes in transmembrane protein concentration and followed a similar trend to that of cell growth, with the highest concentrations in EVs from day 2–4 at an inoculation density of 5000 cells/cm^2^ (Figure 4D). Higher CD105 concentrations could be obtained with constant removal of EVs (i.e., collecting day 2–3 and again day 3–4 as opposed to day 2–4). CD105 yield on a per cell basis was highest at earlier time points (Figure 4E); however, CD105 yield on a per protein basis was increased at high confluence (both higher time points and higher inoculation densities) (Figure 4G).

Angiogenic protein concentrations were measured in lysed EV fractions at different MSC confluence levels and EV collection periods, and were done to align with parallel studies evaluating EV potential for inducing angiogenesis in brain‐derived endothelial cells [17, 30]. Angiogenesis is critically important for recovery following an ischemic stroke or traumatic brain injury, and highly relevant in general to wound healing and tissue repair. The measured concentrations of BMP‐9, EGF, ET‐1, HB‐EGF, leptin, PLGF, and VEGF‐D were under the detection limit. The total concentrations of ANG‐2, follistatin, G‐CSF, HGF, IL‐8, and VEGF‐A were highest on day 2–4 with 5000 cells/cm^2^ inoculation density (Figure 4G). A reduction in bFGF was seen over time in culture. Comparing day 2–3 to 3–4, an increase in G‐CSF, HGF, IL‐8, and VEGF‐A was seen. On a per‐cell basis, higher yields of bFGF, follistatin, and VEGF‐C were found at low confluence, and yields of G‐CSF, HGF, and VEGF‐A were greater at high confluence (Figure S4). A relatively consistent yield of IL‐8 was found, indicating that it was not affected by cell confluence. To better understand protein topology, non‐lysed and lysed EV fractions were measured (Figure S5). All angiogenic proteins showed significantly higher concentrations in lysed samples, indicating a high association of proteins with the EVs.

**FIGURE 5:**
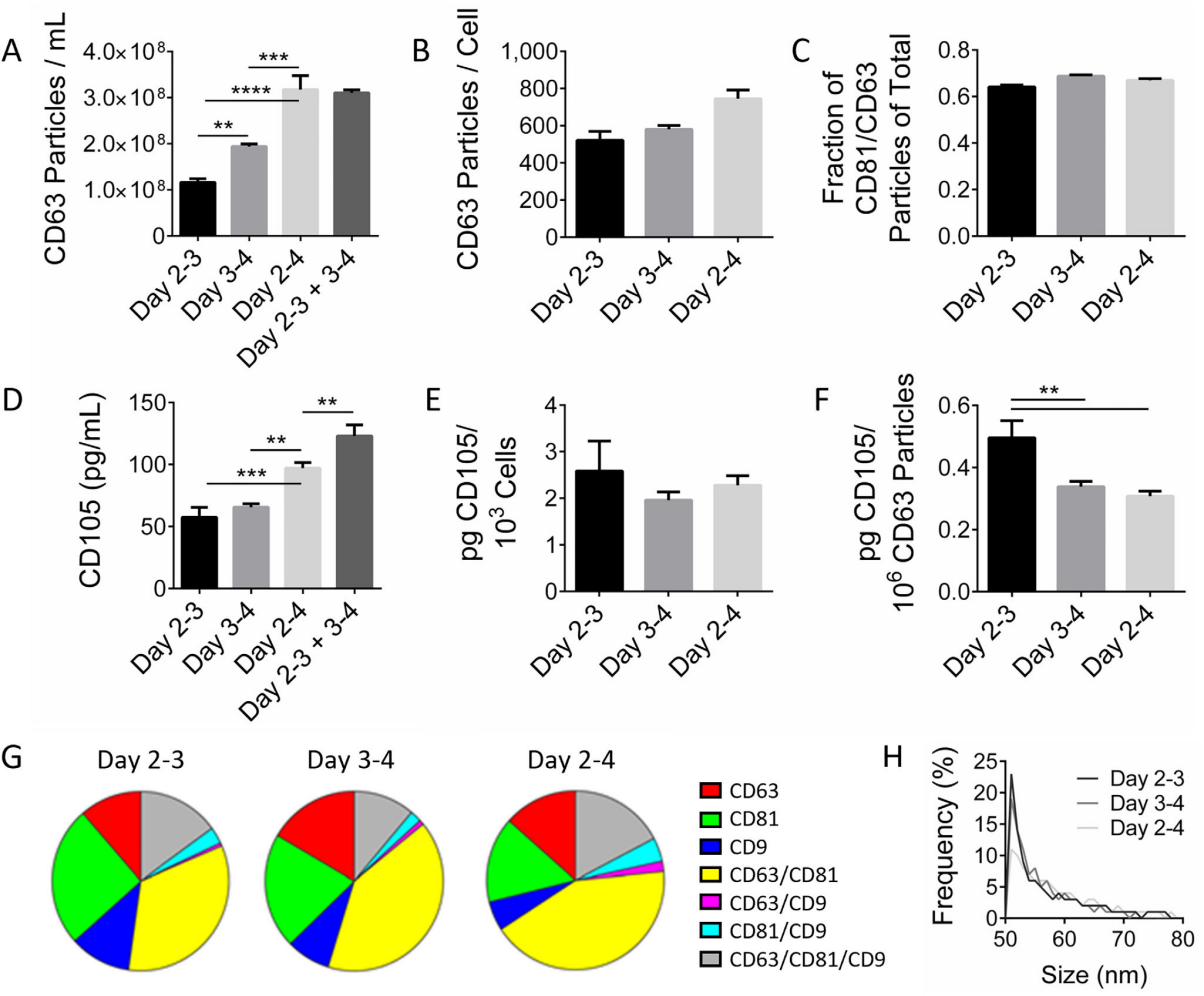
SP‐IRIS and protein analyses of EVs isolated from MSCs at different collection time points (*N* = 3). (A) Total particles bound to CD63 from EV fractions isolated from CM of MSCs collected from day 2–3, day 3–4 or day 2–4. (B) Total particles bound to CD63 measured as a fraction of cell density at time of collection. (C) Fraction of colocalized CD63/CD81 particles of total particles bound to CD63. (D) CD105 concentration in the EV fractions. (E) CD105 content per 10^3^ cells. (F) CD105 content per 10^6^ colocalized particles. (G) Colocalization charts for representative EV samples bound to CD63. (H) Particle size distribution measured by SP‐IRIS.

SP‐IRIS was carried out to confirm changes in EV concentration at different collection time points at the highest 5000 cells/cm^2^ inoculation density, which resulted in the highest EV concentrations as measured by CD105, and the highest angiogenic protein concentrations. Total particle concentrations and co‐localization of EVs isolated in UC medium between days 2–3, 2–4, and 3–4 are presented in Figure 5. Total CD63 particle counts were significantly higher in EVs collected from day 2–4 compared to those collected day 2–3 or 3–4 (Figure 5A). No significant differences in particles/cell or the fraction of co‐localized CD63/CD81 particles were seen (Figure 5B,C). While CD105 and total protein data indicated that more frequent harvest (i.e., collecting day 2–3 and 3–4) would produce the highest total number of EVs based on total protein and CD105 concentrations (Figure 5D), the number of CD63 particles were equal in day 2–4 compared to day 2–3 and 3–4 combined. CD105 concentration measured on a per cell basis was not significantly different, but on per particle basis (Figure 5F), a significant increase in CD105 content/particle was found on day 2–3 as opposed to day 3–4 and 2–4, indicating that CD105 content/particle is higher at lower cell confluence. Each of the EV fractions collected at different time points expressed a similar co‐localization phenotype; however, a higher degree of co‐localization was seen for day 2–4 (Figure 5G). Particle size was consistent across groups (Figure 5H).

## 4 Discussion

With increasing evidence demonstrating the regenerative properties of MSC‐EVs, there is a need to develop clinically applicable production strategies. This study evaluated the use of a SFM to produce MSC‐EVs. PPRF‐msc6 was the first SFM reported in the literature for the successful culture of MSCs where the ingredients used were fully disclosed [15]. It is now widely used for the culture of MSCs from many different sources and has been shown to successfully and reproducibly support the long‐term proliferation of MSCs without compromising their defining qualities [13, 14, 32]. Protocols in this study were chosen to align with commonly used protocols, where MSCs are cultured to approximately 60–80% confluence, followed by culture in EVCM consisting of either UC medium, or the medium prepared without serum (or in this case, serum‐derived components albumin and fetuin) [6, 27, 33]. Analysis of EV populations was done using SP‐IRIS to enable characterization on a single‐EV level via multiplexed phenotyping and counting of individual EVs captured on a microarray through antibodies targeting EV markers [34].

We found a high degree of binding to particles within PPRF‐msc6 when analyzing complete media as opposed to isolated EV fractions. The fact that these particles exhibited no co‐localization of two or more EV‐specific antibodies suggests a lack of EV‐associated particles and outlines the importance of co‐localization analyses to rule out protein contaminants, and analysis of EVs after isolation despite theoretically being able to measure EV content directly from CM. Bias from medium‐derived particles suggested that an EVCM be used from which particles had been removed, but which supported the viability of cells. Removal of particles from PPRF‐msc6 by overnight U did not affect the growth, viability, or surface marker expression of MSCs, supporting the use of UC medium for EV production. The overall higher attainable concentrations of EVs and angiogenic proteins from high confluence MSC cultures without significant changes in EV yield further support the collection of EVs from MSC cultures over a 48 h period.

Despite obtaining higher EV purity when using UC medium for EV collection, EV yield after isolation was reduced. We found that supplementation of UC CM with HSA increased the recovery of EVs during isolation, possibly due to enhanced EV stability, as coating of the ultracentrifuge tubes to prevent adhesion did not have a significant effect. Albumin is often studied for its natural transport properties and enhancement of circulatory half‐life [35], and has been widely used in SFM due to its role as a carrier of biomolecules that support cell growth [36]. It has also been reported to reduce aggregation of proteins [37], which could eliminate concerns related to EV aggregation following freeze‐thaw. Notably, a prior study tested various EV storage conditions and recommended storage at ‐80°C in PBS with the addition of HSA and trehalose [31]. In addition, EVs are surrounded by a protein corona which enhances their function in angiogenesis, skin regeneration, and immunomodulation [38]. It was found that removal of this corona during the EV purification process affected EV functionality, but that this function could be reconstituted through cloaking the EVs with bioactive proteins in albumin [38]. The important role of the corona is further supported by the enhanced uptake of EVs by alveolar cells that were incubated with bovine serum albumin (BSA) [39]. However, it is important to note that the addition of albumin may bias the results of functional assays [40]. For example, BSA‐derived protein aggregates were reported to have protective effects against starvation‐induced apoptosis of rat kidney epithelial cells [40]. Appropriate controls are needed to delineate the functional role of EVs as opposed to other additives in the culture medium or storage buffers.

These experiments further confirmed that the degree of cell confluence influences EV populations. We found increased protein yield at lower confluence, consistent with other studies reporting higher EV yields at low seeding density and low confluence [26, 41]. However, despite a significant increase in protein yield at low confluence, these results did not correlate with single EV analyses. We found no change in CD63+ particle yield over a 24 h period. Similarly, EV collection over a 48 h period resulted in an equivalent number of particles to that of two individual 24 h collections. This contradicts prior reported results which found increased MSC‐EV yield with continuous harvest (two harvests over 24 h compared to one) [26, 41]. The inherent differences between studies are the methods used for analysis. SP‐IRIS measures particle counts specific to EV markers, whereas nanoparticle tracking analysis measures total non‐specific particle count (which may include medium‐derived particles that get consumed at higher cell densities) and ELISA measures protein quantity of lysed EVs, thereby not accounting for changes in surface marker expression of individual particles. It should be noted that the current study did not explicitly evaluate the elimination of apoptotic bodies in collected EV fractions; however, the presence of internal EV marker syntenin‐1 and absence of endoplasmic reticulum marker GRP94 were previously confirmed at the highest inoculation density evaluated (5000 cells/cm^2^) using ExoView cargo analyses [16].

There is still uncertainty as to whether EV quantity is a measure of success in bioprocess development. For example, aged senescent MSCs have been reported to secrete higher numbers of EVs per cell, but lower quantities of protein per EV [42]. The higher EV secretion is hypothesized to be a compensation mechanism by aged MSCs because of a decrease in their protein‐to‐particle ratio [42]. In the current study, changes in protein content within the EV fraction did not align with EV quantity and indicate that protein content per EV changes depending on the culture medium the cells are exposed to and their confluence level. Total protein content, considering appropriate purification strategies to avoid bias of contaminating protein from medium components, as opposed to the number of EVs themselves, may present a more controlled basis for dosage. Regarding angiogenic protein content, it will be important to design EV processes aligned with specific applications, as MSC‐EVs may play dual roles. For example, angiogenic and other proteins or RNAs may enhance oncogenesis and tumor progression in cancer [43], despite playing regenerative roles in other applications such as stroke, wound healing, and tissue repair. Application‐specific screening of different EV sources and manufacturing processes for each application is necessary to ensure safety, and the development of methods to deactivate harmful content may be required.

CD105 is a transmembrane protein present on MSCs and their EVs. CD105 expression has been shown to be altered by culture medium [44, 45], differentiation [46], time in culture [47], and cell density [48]. Studies have demonstrated a significant positive correlation between MSC CD105 levels and enhanced chondrogenesis [49, 50] and cardiac regenerative potential [51], although a subpopulation of CD105‐negative MSCs has been found to have higher immunomodulation capacity compared to their CD105‐positive counterparts [52]. We have previously reported a positive correlation between CD105 concentration in the EV fraction and its ability to induce angiogenesis (tube formation) of brain endothelial cells [30]. In the current study, the concentration of CD105/CD63+ particles was significantly higher for EVs isolated from culture in UC medium compared to PPRF‐msc6 and harvested on day 3 as opposed to day 4. The reduction of MSC CD105 expression in PPRF‐msc6 medium on day 4 aligns with prior studies reporting reduced CD105 expression with increased cell density [48]. However, it was interesting to see that the use of UC medium inhibited this downregulation. It is possible that a component within PPRF‐msc6 contributes to CD105 downregulation at high confluence and is removed by UC, which could explain the consistently higher CD105 content in UC EV fractions compared to PPRF‐msc6 EV fractions. Notably, the culture of MSCs in SFM has been associated with reduced CD105, without affecting their differentiation ability or immune function [44]. Studies have also reported that MSCs lack CD105 expression prior to in vitro culture [48]. Therefore, the role CD105 plays in the regenerative properties of MSCs and their EVs is currently unclear and requires further investigation.

The current study utilized differential UC due to its high use throughout the literature and availability in most laboratories. UC has inherent limitations such as co‐isolation of protein aggregates and lipids, aggregation and damage of EVs, and scalability. Different isolation methods could produce EV fractions with differing proteomic profiles due to interactions that could occur during UC. However, limitations exist for all current EV isolation techniques, and protein contamination by SFM components has also been seen in higher purity techniques such as ultrafiltration‐size exclusion liquid chromatography [20]. Similarly, while this study evaluated EVs isolated from MSCs cultured in static T‐flasks, T‐flasks are not amenable to large‐scale processes. Scalable culture methods in bioreactors enable higher numbers of cells to be produced in a single batch, while offering a more homogeneous environment and higher nutrient and oxygen transfer rates [16]. These systems expose MSCs to different culture conditions and forces such as fluid shear can influence growth rates and gene expression, thereby altering EV composition [16]. Time of collection, as we have defined for static systems, is not directly applicable to other culture platforms, or for cells isolated from different patients or sources; however, the analysis provided illustrates the importance of considering this parameter in the development of EV‐related bioprocesses. The experimental procedures outlined in this study should be applied to different isolation and production methods to support EV manufacturing efforts. Finally, further studies are needed to highlight the functional consequences of EV compositional differences to better understand their mechanism of action and the overall impact of bioprocessing parameters.

## 5 Conclusions

MSC‐EVs offer a promising treatment in applications of regenerative medicine. However, inconsistency in cell culture and EV collection protocols serves to undermine the use of EVs in clinical settings. This study found that medium deprived of essential nutrients, such as albumin, significantly reduced MSC viability and MSC‐specific surface marker expression over a 24 h period, while medium ultracentrifuged to reduce contaminating proteins was able to maintain viability and MSC properties, while improving the purity of isolated EV fractions. It was further found that MSC confluence alters the proteomic profile of EVs. This study outlines the importance of considering the effects of EV collection medium on MSC properties and their subsequent EV populations, as well as how medium components may bias the results of EV characterization. These considerations have a large impact on the EV field and contribute to the standardization of EV production and collection protocols to improve reproducibility in translational studies that can enhance the clinical utility of MSC‐EVs.

## Author Contributions

**J.P**.: conceptualization, data curation, formal analysis, methodology, visualization, writing – original draft, writing – review and editing; **S.H.T**.: writing – review and editing; **N.A.D**.: supervision, writing – review and editing; **A.S**.: conceptualization, supervision, funding acquisition, writing – review and editing.

## Funding

This work was supported by the Natural Sciences and Engineering Research Council of Canada under grant number RGPIN‐2019‐07196.

## Ethics Statement

This study was conducted in accordance with the Declaration of Helsinki and informed consent was obtained from all subjects involved in the study. Human ethical approval was obtained from the University of Calgary Health Research Ethics Board (ID: REB15‐1005).

## Conflicts of Interest

The authors declare no conflicts of interest.

## Supporting information




**Supporting File**: biot70264‐sup‐0001‐SuppMat.docx.

## Data Availability

All data generated or analyzed during this study are included in this published article and Supporting Information files.
