# Supplementary material for: The Impact of Collection Protocol on the Yield and Purity of Mesenchymal Stem Cell‐Derived Extracellular Vesicles Isolated From Serum‐Free Media
Source: Biotechnol J. 2026 Jul 2;21(7):e70264. doi: 10.1002/biot.70264 (PMC13325519; doi:10.1002/biot.70264)
Supplement: Supplementary file 1 — Supporting File: biot70264‐sup‐0001‐SuppMat.docx. [file BIOT-21-e70264-s001.docx]

# Supplementary Methods

## Ultracentrifuge tube coatings

25 mL polycarbonate ultracentrifuge tubes (Beckman Coulter, Brea, CA) were utilized for EV isolation with or without coating. For HSA coated tubes, tubes were incubated with HSA (InVitroCare Inc., Frederick, MD) at a concentration of 10 mg/mL in DPBS for 1 h at room temperature, and then washed 5 times with DPBS immediately prior to use. For Sigmacote coated tubes, Sigmacote (Sigma-Aldrich, St. Louis, MO) was distributed over the tube surface and tubes were left to dry overnight. Tubes were then washed 5 times with DPBS prior to use.

# Supplementary Data

Table S1. Antibodies used for flow cytometry analysis of MSCs

| Antibody | Clone | Manufacturer |
| --- | --- | --- |
| Mouse Anti-Human CD90 IgG1 FITC | F15-42-1 | Serotec (Bio-Rad) |
| Mouse Anti-Human CD105 IgG2a FITC | MEM-229 | Abcam |
| Mouse Anti-Human CD14 IgG2a | TUK4 | Serotec (Bio-Rad) |
| Mouse Anti-Human HLA DR IgG3 PE | HL-39 | Serotec (Bio-Rad) |
| Mouse IgG1 FITC (isotype control) | W3/25 | Serotec (Bio-Rad) |
| Mouse IgG2a FITC (isotype control) | OX-34 | Serotec (Bio-Rad) |
| Mouse IgG3 PE (isotype control) | n/a | Serotec (Bio-Rad) |


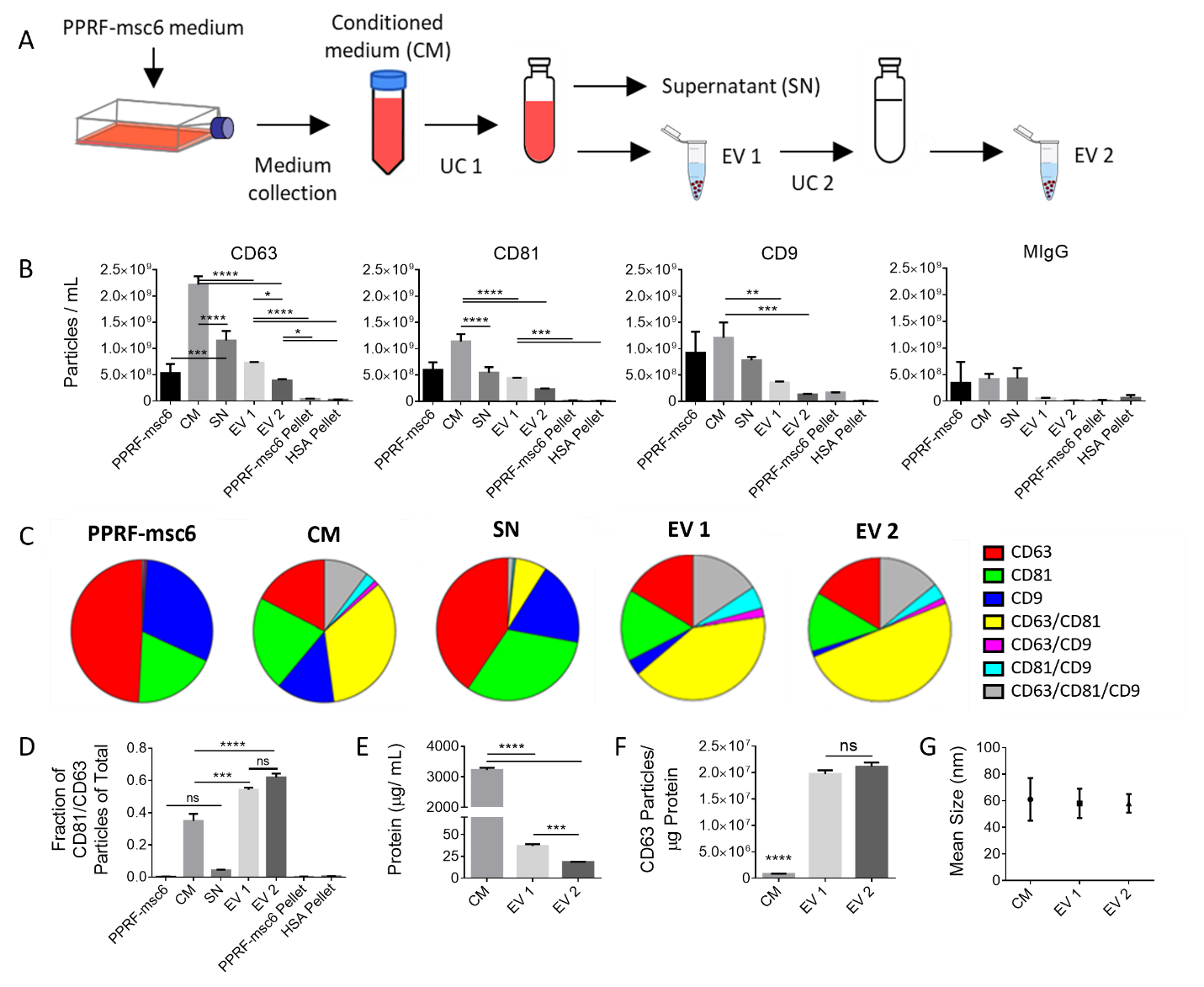


Figure S1. SP-IRIS and protein analyses of CM and EV fractions collected from MSCs cultured in PPRF-msc6 medium after 1 or 2 stages of ultracentrifugation (UC). A) Schematic of the process used for producing and isolating EV fractions. B) Total particles bound to CD63, CD81, CD9, and MIgG antibodies on tetraspanin microarray chips. Controls include fresh PPRF-msc6 prior to utilization with cells (PPRF-msc6), the pellet of fresh PPRF-msc6 diluted 1:1 with DPBS following UC (PPRF-msc6 pellet), and the pellet of fresh 2% HSA in DPBS following UC (HSA pellet). Volumes of media undergoing UC and volume of DPBS for resuspension for controls for all conditions were equivalent. Percentages above bars for EV fractions represent apparent recovery of particles from CM. C) Co-localization charts for representative samples bound to CD63. D) Fraction of colocalized CD63/CD81 particles of total particles bound to CD63. E) Total protein measured in CM and EV fractions. F) Total CD63 bound particles per µg total protein measured in CM and EV fractions. G) Mean size and standard deviation plotted for particles in CM and EV fractions as measured by SP-IRIS.


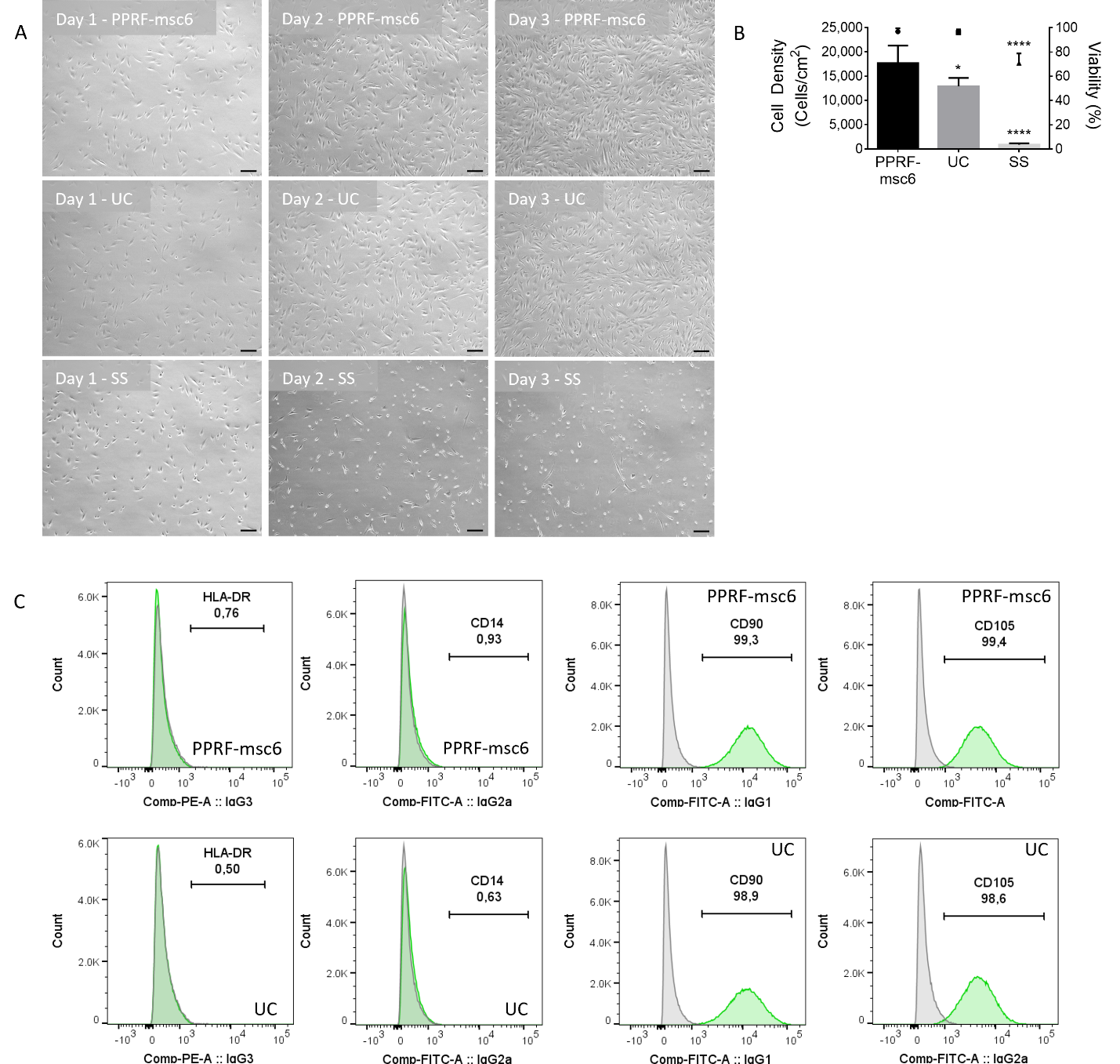


Figure S2. Comparison of MSCs cultured in PPRF-msc6 medium, ultracentrifuged PPRF-msc6 (UC) medium, and PPRF-msc6 medium without HSA and fetuin (SS) for 3 days. A) Photomicrographs of MSCs cultured in PPRF-msc6 medium, UC medium, and SS medium from day 1 to day 3. Scale bar = 100 µm. B) Cell density and viability of harvested MSCs from day 3 cultures. Error bars represent SD. ****P <0.0001. C) Flow cytometry analysis for markers HLA-DR, CD14, CD90, and CD105 for MSCs cultured in PPRF-msc6 medium and in ultracentrifuged PPRF-msc6 (UC) medium for 3 days. Grey represents control and green represents experimental conditions for all samples.

Figure S3. SP-IRIS and protein analyses of EV fractions isolated from day 4 MSCs in coated UC tubes (A-D) and in different EV isolation media (E-H). A) Total particles bound to CD63 antibodies on ExoView tetraspanin chips for EVs derived from UC CM isolated in UC tubes with no coating, coated with Sigmacote, and coated with HSA. B) Total protein measured for all UC tube coating conditions. C) Fraction of colocalized CD63/CD81 particles compared to total bound to CD63. D) Mean size and standard deviation plotted for each condition as measured by SP-IRIS.



Figure S4. Angiogenic protein yield (protein/cell) in EV fractions at different inoculation densities and harvest time points. All concentrations are normalized to per mL of conditioned medium.


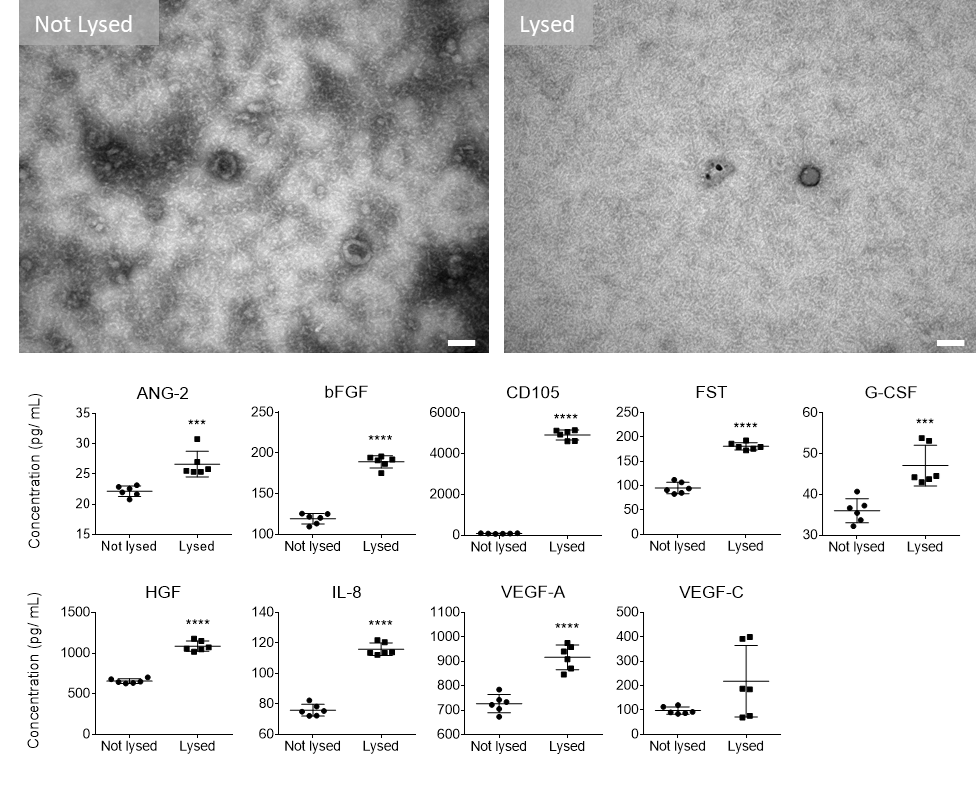


Figure S5. Comparison of non lysed and lysed EV fractions isolated from MSCs. Top) TEM images of non-lysed (left) and lysed (right) samples imaged at 80 kV. Scale bar=100 nm. Bottom) Luminex analyses of angiogenic proteins from non-lysed and lysed EV fractions. EVs were lysed by suspension of the EV pellet in 1x RIPA buffer with 10 µL/mL protease inhibitors. Statistical significance relative to non-lysed samples.
